# Differential Effect of Formality of Speech Style on Negation Selection in Korean Children and Adults

**DOI:** 10.1007/s10936-026-10290-5

**Published:** 2026-09-06

**Authors:** Suji Jung, Jieun Kiaer, Naya Choi

**Affiliations:** 1 Future Education and Care Research Team, Korea Institute of Child Care and Education, Seoul, South Korea; 2 Faculty of Oriental Studies, University of Oxford, Oxford, UK; 3 Department of Child and Family Studies, Seoul National University, Seoul, South Korea

**Keywords:** Korean negation forms, Acquisition of negation, Long-Form Negation (LFN), Short-Form Negation (SFN), Formality of speech styles

## Abstract

Korean negation has two forms, Short-Form Negation (SFN) and Long-Form Negation (LFN), claimed to have different pragmatic meanings in terms of formality. This paper investigated whether Korean 5-year-old children and adults responded to the level of formality in a conversation, using an experimental design in which a puppet asked the participants either formal or informal-style questions. The results showed that adults produced long-form negation significantly more often in the formal condition than in the informal condition, whereas children predominantly preferred short-form negation across both conditions. Generalized linear mixed-effects modelling revealed significant main effects of speech style and age group. Although the interaction between speech style and age group was not statistically significant, planned pairwise comparisons indicated a significant effect of speech style in adults but not in children. These findings suggest that sensitivity to speech style in negation selection is evident in adults but is not yet fully established in 5-year-old children, supporting the view that pragmatic control over negation forms continues to develop during language acquisition.

## Introduction

This study aims to examine whether two forms of Korean negation, Short-Form Negation (SFN) and Long-Form Negation (LFN), are produced in response to formality of speech style in the context of verbal conversation. Additionally, the study examines if there is a developmental difference in the relationship between speech style and negation form in the utterances of young children as opposed to adults. It has been debated whether SFN and LFN in Korean are different in pragmatic meaning, especially in terms of formality (Han, 2014; Park, 2007; Sohn, 2013). Additionally, the meanings of negation forms can be distinguished in terms of politeness (Han, 2014; Sells, 2015; Sohn, 2013). Koreans shift speech styles to show different indexical meanings such as formal presentational stance and emotionally unrestrained and casual situations (Brown, 2015; Yun, 2000). Therefore, it is expected that speech styles can form a communicative context which the listeners are demanded to have a polite or casual attitude, leading to more frequent uses of one of the two negation forms. If this relationship between the formality of speech level and negation forms presents itself in adults, it would additionally be worth investigating if young children are also sensitive to the formality of a communicative situation in choosing the more suitable form of negation.

Korean negation needs to be studied with the focus of the contexts for formal and informal attitudinal changes to reflect the cultural and linguistic features of Koreans’ communication. When communicating in Korean, the context in which the message is delivered, as well as the message itself, is considered crucial in communication. Due to this cultural feature, Koreans’ communication is classified as a High Context (HC) communication based on the high level of impact of context on communication and language (Gudykunst & Nishida, 1986; Kim et al., 1998; Sak & Shaw John, 2005). Additionally, as Korea was once a Confucian country and has inherited Confucian values, politeness and vertical hierarchy are respected among members of society until now (Brown, 2011). Based on these cultural and linguistic features of Korean communication, this study tested whether Korean adults and children chose a particular negation form depending on formality, comparing their production of SFN and LFN in the contexts of formal and informal speech styles.

## Two Forms of Korean Negation and Their Differences

Korean has two different forms of negation, Short-Form Negation (SFN) and Long-Form Negation (LFN) (Sells, 2015). It is generally agreed that Korean negation can be divided into two forms by its length (Lee, 1993; Noh et al., 2013). Negation is either expressed by placing the negative morpheme “an (i)” in front of the verb in SFN or by attaching negative morpheme “-(ci) anh-” after the verb in LFN (Hagstrom, 1997). An affirmative sentence (1) can be negated in two forms ((2) SFN, (3) LFN):

1. Affirmative sentence: kom-i sa-kwa-lul mek-nun-ta. (A bear eats an apple.)
2. Short-Form Negation: kom-i sa-kwa-lul an mek-nun-ta. (A bear does not eat an apple.)
3. Long-Form Negation: kom-i sa-kwa-lul mek-ci anh-nun-ta. (A bear does not eat an apple.)

Short-Form and Long-Form Negation were considered to be synonymous in some literature (Park, 1972, 2007). It also has been demonstrated that the difference of the two forms of negation lies in the scope of interpretation (Cho, 1975; Kim, 1975; Kuno, 1980). However, evidence from recent studies has shown that the meaning of SFN and LFN can be differentiated, implying that they cannot be used randomly or interchangeably in every communicative situation. It has been argued that there may be a more complex meaning to be inferred from Long-Form Negation compared to Short-Form Negation. Suffix “-ci” in Long-Form Negation seems to demand a more complex cognitive burden in processing compared to Short-Form Negation. Specifically, this burden was asserted in research supporting relevance theory (Choi, 1988; Sperber & Wilson, 1986; Wilson, 2000), which emphasizes an addressee’s active inference process using his/her expectation of an utterance’s relevance in addition to literal meaning in interpreting meaning. According to the studies, the proposition preceding suffix “-ci” in a Long-Form Negation sentence shows the meta-linguistic use of language to represent additional meaning (Noh, 2000). As pragmatic enrichment is necessary for the meta-representational elements to contribute to the truth-conditional contents (Noh, 2016), more pragmatic inferences can be made to enrich the meaning of LFN with suffix “-ci” compared to SFN.

Furthermore, past research has shown that more complex meanings relating to contextual information can be drawn from LFN than SFN. The two negation forms can be distinguished by whether they can be used as descriptive negation (DN) or meta-linguistic negation (MN) (Noh et al., 2013). Previous studies supporting relevance theory showed that Short-Form Negation could also be used as MN and be interpreted through a pragmatic inference process if contextual information is sufficiently provided (Carston & Noh, 1996; Lee, 2005; Noh et al., 2013). Thus, these results suggest that, in the absence of such supplementing contextual information, more complex contextual meaning can be elicited from LFN itself than from SFN itself.

It is supposed that the more complex and contextual meaning of LFN could be attitudinal meaning, given that LFN and SFN can also be differentiated in terms of politeness. Using LFN could show a politer attitude (Han, 2014; Sohn, 2013), while SFN could be considered as more straightforward and casual (Sells, 2015). Han (2014) analyzed a corpus and found that SFN was used to negate the information presented by a conversational partner more firmly and conclusively. Meanwhile, LFN was used to show an addresser’s polite attitude by expressing negation in a roundabout way. As conclusive negation can be considered disrespectful (Givón, 1993); the addresser can use Long-Form Negation to avoid the burden of opposing an addressee’s presumption directly, and thus be considered polite (Han, 2014). These subtle differences in the pragmatic meaning of the two negation forms enable speakers to communicate richer and more elaborate meanings.

In summary, adequate recent evidence indicates that there are semantic and pragmatic differences between SFN and LFN. However, little work has been done empirically to verify if attitudinal meanings of being polite or casual are differentially embedded into SFN and LFN in Korean adults and children.

### Development of Korean Negation in Young Children

Previous research on negation development in English speaking children have shown that children can produce negation from the second year of life (Bloom, 1970; Pea, 1980) and that the order of semantic development of negation of Korean children is similar to that of English-speaking children (Choi, 1988; Kim, 1997).

However, Korean children produce Long-Form Negation later than Short-Form Negation. According to comprehensive analysis on results from recent studies, it was found that Korean children use the SFN morpheme “an” from 1.11 to 2.5 years and do not use LFN until 2.11–4 years (Kim, 2014). A similar result was found that the negative adverb of SFN “an” first appeared in children’s utterances from 20 to 21 months, whereas the auxiliary verb of LFN “-(ci) ani-hada” was found in children’s utterances from 28 to 31 months (Chang et al., 2014; Lee & Im, 2003). Choi and Zubin (1985) found that LFN appeared in children’s utterances from later than three years and five months. Cho and Hong (1988) showed that two-year-olds could understand but could not repeat LFN sentences. Similar to English-speaking children who have difficulty in processing negative sentences (Nordmeyer & Frank, 2018), Korean-speaking children also find it difficult to process negative expressions. In other words, they struggle to process LFN more than SFN.

The tendency that Korean children acquire SFN first and LFN later may result from the fact that the latter is more complex than the former in terms of syntactic, semantic, and pragmatic aspects. Kim (2014), in analyzing longitudinal acquisition of negation of young Korean children, reported that two-year-olds replaced LFN with SFN even in the contexts where LFN was more appropriate. The author referred to this phenomenon as “overgeneralization of short-form negation”. She explained that SFN enabled children to communicate effectively due to its shortness of production and clarity in meaning, thus children frequently used it.

In this study, we aimed to investigate the depth of young children’s semantic understanding of LFN, supposing that young children may have difficulty in using it because of the embedded attitudinal meanings of politeness.

### Relationship Between Formality as Pragmatic Context and Choice of Negation Form

Context plays a powerful role in processing negative sentences (Nordmeyer & Frank, 2018). Previous studies have shown that the processing of negative sentences was sensitive to linguistic contexts (Dale & Duran, 2011; Giora et al., 2007; Lüdtke & Kaup, 2006), visual figures (Wason, 1965), and visual information (Nordmeyer & Frank, 2018).

Considering the cultural and linguistic features of Koreans’ communication, it is possible that the formality or informality of a situation, indexed by verbal endings, can constitute a pragmatic context and thus influence the choice of negation form. In Koreans’ communication, the context has a great influence on language and communication (Kang, 2018), which is consistent with the traits of Hall (1976)’s High-Context (HC) culture.

Hall (1976) proposed the concept of High Context (HC) and Low Context (LC) culture and argued that the level of context of a culture has an influence on language and communication. According to Hall, cultural orientation can be explained as a continuum, with High Context (HC) and Low Context (LC) on either end (Kim et al., 1998). Previous studies have compared Koreans’ and Americans’ communication styles representative of HC and LC communication respectively. They consistently found that Koreans used more implicit and indirect messages, and the context and social relationship between interlocutors played more important roles in communication compared to Americans (Kang, 2018; Gundykunst et al., 1996). Though the previous studies which classified Koreans’ communication as HC communication can be limited in relying on subjective analysis, it is generally agreed that the social context in communication, especially interpersonal relationship, is more likely considered as a priority in choosing a proper form of language in Koreans’ communication compared with LC communication (Chen, 2017). Given that the level of formality of context in communication is expressed through the different speech styles in Korean (Yun, 2000), it is possible that the relative difference in formality between speech styles can lead to the use of different forms of negation to index more or less indirectness and soften Koreans’ communication.

Korean has distinctive speech styles, and each speech style is expressed with different verb-endings (Lee, 2009). Korean speech levels reflect a distinctive feature of Korean interpersonal communication: the need to show an appropriate attitude toward an addressee based on the relative social ranking of the interlocutors and the communicative environment. Koreans use code-switching of speech styles to achieve communicative goals, encoding power, solidarity (different degrees of intimacy and formality of the situation), respect, and obedience, thus showing a speaker’s attitude toward the listener and conversational situation (Yun, 2000).

Previous research has generally agreed that Korean has six speech styles (Brown, 2015; Sohn, 2013; Yeon & Brown, 2013). The names of the six speech styles and verbal endings in Korean are as follows:

1. formal style/“hapsyo-chey”: -supnita
2. polite style/“hayyo-chey”: -yo
3. semi-formal style/“hao-chey”: -so
4. familiar style/“hakey-chey”: -ney
5. half-talk or intimate style/“panmal”/“hey-chey”: -e
6. plain style/“hayla-chey”: -nunta

Among the six speech styles listed above, only four speech styles—formal, polite, half-talk, and plain styles—are commonly used by modern Korean people (Brown, 2015; Kim, 2022). Semi-formal and familiar styles can be found in Korean historical dramas (Kim, 2022). Additionally, as the half-talk and plain styles are commonly used in combination and differentiated in terms of discourse function,^1^ in practice, modern Korean speech styles include three styles: (1) formal style, (2) polite style, (3) mixed style of half-talk and plain style (Brown, 2010, 2015). On the other hand, these speech styles of modern Korean are normatively classified into honorific and non-honorific styles: honorific styles include formal and polite styles, while non-honorific styles include half-talk and plain styles.

However, previous studies have repeatedly argued that these four speech styles of Korean are differentiated in various feature values other than “honorifics”. Specifically, “formality”, “being used for adults”, “gender”, and “information status” can differentiate speech styles (Brown, 2015; Yun, 2000). As for “formality”, the sentence endings of the formal and polite styles, *-supnita* and *-yo* are both used toward older or superior persons as honorific styles, but two sentence endings are differentiated in terms of formality: *-supnita* indexes formality, while *-yo* indexes informality (Brown, 2010, 2015; Yun, 2000). As an informal honorific style, the sentence ending *-yo* is used to show the expression of affect which is avoided in formal communicative situations. For these reasons, Brown (2010) called the polite style “the middle ground style” between the polarities of honorific and non-honorifics style.

The different speech levels can reflect a pragmatic context in which speakers are required to take a polite or casual attitude, which can be linked to different meanings of SFN and LFN. Han (2014) claimed that LFN was preferred to SFN in formal situations. It was argued that LFN can soften the intensity of negating information and thus, due to its pragmatic function of politeness, LFN could be more suitable for formal situations. Sohn (2013) also suggested that there is a slight stylistic difference among the two forms where SFN is less formal than LFN.

If the formality of speech level can have effects on the selection of a negation form in adults, it is worth investigating whether young children who can produce both speech levels and forms of negation are also sensitive to formality as context. Research on the acquisition of Korean speech levels has shown that young children can express various speech levels, including honorific verbal endings, from two to three years (Lee, 2009; Lee et al., 2013). Five-year-old children seemed to use both the formal and informal speech levels, as well as both forms of negation, raising the question of whether they can relate speech levels and negation forms. Although LFN was not frequent in young children’s utterances in previous studies (Lee, 2009; Kim, 2003, 2014), there is a possibility that production of LFN can be increased if experimental design provides children with a sign for a polite attitude conducive to producing that negation style. If 5-year-olds were able to choose to produce a negation form informed by the context in the same way as adults, this could suggest that, not only negative propositional meaning, but also pragmatic knowledge related to the use of negation are acquired from the earliest stage of negation development. On the other hand, if children did not distinguish the negation forms based on context, this could indicate that the complex semantic and pragmatic meanings of negation styles are acquired and used later than negative propositional meaning. However, little empirical research has been done thus far to explore the influence of speech level on negation selection in Korean children and adults. Speech styles can be the context for attitudinal change, leading to the production of one negation form to index a formal or casual stance. Therefore, in this study, we compared Korean adults’ and children’s choices of negation forms in the contexts of formal and informal style levels.

### Present Study

This study aimed to verify if formal or informal verbal endings can provide contexts where adults and children prefer using one of two forms of negation. Among the commonly used speech styles in modern Korean, we selected the formal and polite speech styles to represent the “formal condition” and the “informal condition” respectively.

There were three reasons for choosing formal and polite styles to represent formality and informality of experimental conditions respectively. First, to verify the effect of “formality” of speech styles on negation selection, the effect of value feature “honorifics” should be controlled by selecting two speech styles, both honorific or non-honorific. Second, since the half-talk style (a representative informal speech style) has pragmatic meanings of a socially close relationship in addition to informality (Kim, 2022), formal and polite styles, which denote a socially distant relationship were more appropriate for the experimental situation in which a participant has a conversation with a stranger (a puppet) whose age is unknown. In Korean children’s utterances, the half-talk style is only used in conversation with other children of the same or younger age and intimate family members. Third, formal and polite styles are honorific styles representative of the formal and informal styles respectively in contemporary Koreans’ utterances (Brown, 2010).

The specific research questions were as follows:

1. Is there a difference in the choice of negation forms in Korean adults and young children depending on experimental condition (formal vs. informal)?
2. Is there a differential effect of formality of speech style on the choice of negation forms between Korean young children and adults?

After building up the baseline of reaction in adults, the same task was given to 5-year-old children to contrast their production of negation. Our first hypothesis was that the formal and informal speech styles as pragmatic context could prompt participants to adopt a politer or more casual attitude respectively, leading them to in turn produce SFN or LFN more. The second hypothesis was that this tendency can be different in children and adults.

## Method

### Participants

35 adults and 31 children who were monolingual native Korean speakers and have lived in South Korea participated in the experiment. Adult participants, 17 males and 18 females, were recruited from two universities in Seoul and Suwon and a corporate research institute in Daejeon. The ages ranged from 19 to 36 with the mean age of 25.41 ± 4.16. Their occupations were undergraduate students, graduate students, administrative staff in a university, and office workers. One participant who was 48 years old was classified as an outlier in terms of her age and thus not included in the final sample. All participants were highly fluent in standard Korean and used it in daily circumstances. Participants were randomly placed in one of two experimental groups: 19 participants were placed in a formal speech style condition and 16 were allocated to an informal speech style condition.

Child participants, 19 boys and 12 girls, were recruited from a kindergarten in Seoul, Korea. They were all 5 years old (Mean = 66.26 months, range = 60–71 months). They were recommended by head teachers of the kindergarten as healthy and without any developmental issues. Two children were not included in the final sample because of atypical articulation and silence due to shyness. Children were randomly divided into two experimental groups: 16 children participated in a formal condition and 15 children took part in an informal condition.

### Stimuli

The experiment assumed a situation where participants had a conversation with a female puppet, “Kong-kong-ee”. A Bluetooth speaker connected to a laptop was placed inside the puppet. The voice of the puppet and pictures of various animals were presented using PowerPoint slides. The voice of a twenty-nine-year-old female adult who speaks standard Korean was recorded for experimental stimuli. We asked her to read the sentences in a neutral way so that children and adults both feel a similar degree of friendliness to the puppet as a conversational partner. To confirm the validity of stimuli, we gave a draft version of the recorded voice to two Korean graduate students to get feedback. We asked them about if the speaker used a neutral voice that was not biased between children and adults and if there was any difference in intonation and attitude of the speaker between formal and informal conditions. Reflecting on the feedback, we recorded the voice used for experimental stimuli.

The puppet introduced herself as a blind person and asked participants to answer her questions about the pictures. The puppet requested simple descriptions to inform her of whether her assumptions about the pictures were right or wrong. Using puppets with portable speakers can create a natural one-to-one conversation setting to elicit utterances similar to a spontaneous conversation from participants. In addition, this can naturally provide participants with the pragmatic information we intended to manipulate in experimental conditions. Given these effects, previous studies have also used the same paradigm to investigate children’s inferences over speakers’ pragmatic intention (e.g., Kurumanda, 2013).

This experiment used a between-group design where there were two experimental conditions (formal and informal) within each experimental group (children and adults). In the formal speech style condition, the puppet spoke with formal style verbal endings (-supnita, -nikka) constantly. In the informal speech style condition, the puppet produced sentences with informal style verbal endings (-yo) until the end of the experiment. As an example, the question “Does the tiger put on clothes?” was phrased “ho-lang-i-ka os-ul ip-sup-ni-kka?” in the formal condition but “ho-lang-i-ka os-ul ip-e-yo?” in the informal condition. These two sentences have the same propositional meaning (Table 1). The expected answers can be classified into four types: (1) formal style SFN, (2) informal style SFN, (3) formal style LFN, (4) informal style SFN (Table 2).

The puppet also produced filler questions consisting of various types of questions including Wh-questions. Verbal endings of fillers were varied in two conditions in the same way as in experimental questions. Each participant was randomly placed in one of two experimental conditions and presented with one of five lists of items in which the order of items was partly counterbalanced.

**Table 1:**
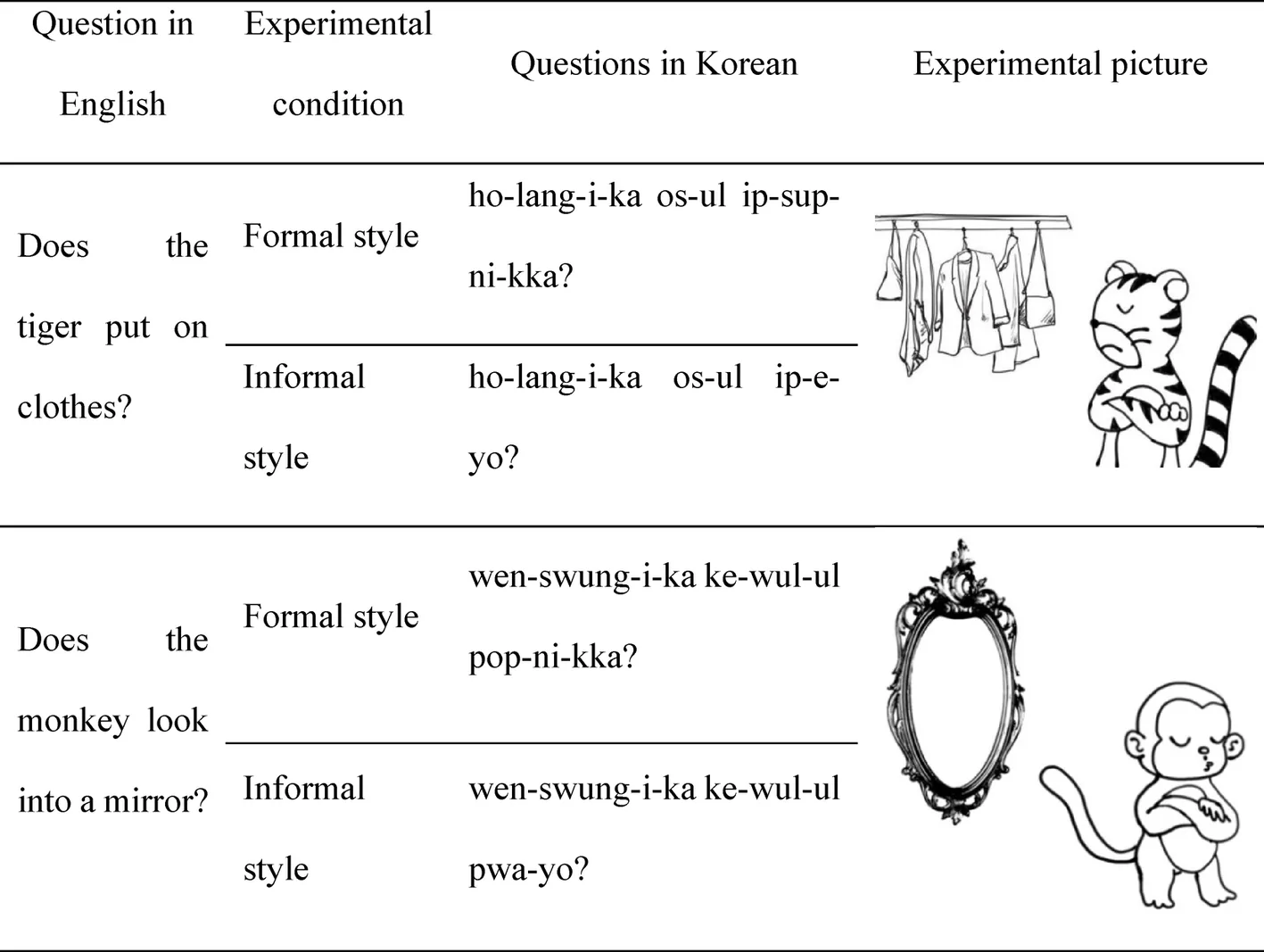
Examples of questions in English and Korean and experimental pictures

**Table 2:** Examples of target responses in English and Korean

Target response in English	Types of response	Target responses in Korean
No, the tiger does not put on clothes.	Formal style SFN	a-ni-yo. ho-lang-i-ka os-ul an ip-sup-ni-ta.
	Informal style SFN	a-ni-yo. ho-lang-i-ka os-ul an ip-e-yo.
	Formal style LFN	a-ni-yo. ho-lang-i-ka os-ul ip-ci anh-sup-ni-ta.
	Informal style LFN	a-ni-yo. ho-lang-i-ka os-ul ip-ci anh-a-yo
No, the monkey does not look into a mirror.	Formal style SFN	a-ni-yo. wen-swung-i-ka ke-wul-ul an pop-ni-ta.
	Informal style SFN	a-ni-yo. wen-swung-i-ka ke-wul-ul an pwa-yo.
	Formal style LFN	a-ni-yo. wen-swung-i-ka ke-wul-ul po-ci anh-sup-ni-ta.
	Informal style LFN	a-ni-yo. wen-swung-i-ka ke-wul-ul po-ci anh-a-yo.

All pictures used in the experiment described an animal doing something toward/with objects. To be specific, the pictures which targeted negative expression depicted an animal being not interested in objects (Table 1). For example, participants were asked to answer an experimental question looking at a picture of a bear with arms crossed, eyes closed, and lips downturned. On the other hand, filler pictures depicted animals taking various actions with a neutral attitude. There were three pictures for a warm-up session and 20 pictures for an experimental session of which half were fillers.

### Procedure

Participants were invited into a quiet room for the experiment. The experimenter introduced participants to the puppet and asked them to listen to the puppet’s instructions and answer the puppet’s questions. The experiment consisted of two parts: a warm-up session and an experimental session. In the warm-up session, participants were trained to respond in full sentences with SOV structure, as it was shown that participants tended to simply answer “no” to questions without training in a pilot test. The questions in the warm-up session targeted positive description without negation, which was the same as the filler questions in the experimental session. In the experimental session, participants were presented with 20 questions. Of these, half were experimental questions that targeted negative descriptions and the other half were filler questions that targeted positive responses. Due to the filler questions, all participants were highly confused about what the experiment was for after finishing. The confusion indicates they were blind to the research hypotheses and their responses during the experiment were not conscious of these.

When child participants hesitated to answer the question from the puppet or produced a positive sentence in answering a question which targeted negative description, an experimenter asked him/her the same question again up to three times. When a child did not produce a targeted sentence after repeating the question three times, the response was evaluated as an unclassifiable response and excluded from statistical analysis.

An experiment took about three to four minutes for adults and six to seven minutes for 5-year-olds to complete. After all procedures finished, coffee vouchers were given to adult participants and crayons to child participants. The verbal speech of participants was audio-recorded in full and transcribed after the experiment.

### Statistical Analysis

For statistical analysis, sentences in participants’ speech were coded based on two criteria: level of formality and type of negation. Specifically, each sentence participants produced was classified as being either formal or informal in speech style and representing either long or short negation. Children made various types of unclassifiable responses which were excluded from analysis. For negation forms, the types of unclassifiable responses were yes/no answers without full sentences, positive statements, nothing answered, and grammatically incorrect sentences. For sentence endings, if sentences ended without endings such as “-da” and “-yo”, they were coded as unclassifiable.

Statistical analyses were conducted in R (version 4.2.2). To account for the repeated-measures structure of the data, we fitted generalized linear mixed-effects models (GLMMs) using the glmer() function in the lme4 package (Bates et al., 2015). The dependent variable was the type of negation produced (long-form vs. short-form negation), which was analyzed using a binomial logistic model with a logit link function. Fixed effects included speech style condition (formal vs. informal), age group (adults vs. children), and their interaction. Random intercepts were included for participants (ID) and test items (question number, QN) to account for repeated observations within participants and variation across items. Model coefficients are reported as regression estimates (β), standard errors (*SE*), Wald *z*-statistics, and associated *p*-values. To evaluate whether the interaction between speech style and age group significantly improved model fit, we additionally conducted a likelihood-ratio test (LRT) comparing models with and without the interaction term. Following the generalized linear mixed-effects analysis, planned pairwise comparisons were conducted using estimated marginal means implemented in the emmeans package (Lenth, 2024) to examine the effect of speech style separately within each age group. *P*-values were adjusted using the Tukey method.

## Results

### Negation-Form Production

To examine how adults and 5-year-old children selected negation forms across speech-style conditions, the frequencies of long-form negation (LFN) and short-form negation (SFN) were compared between the formal and informal conditions.

**Fig. 1:**
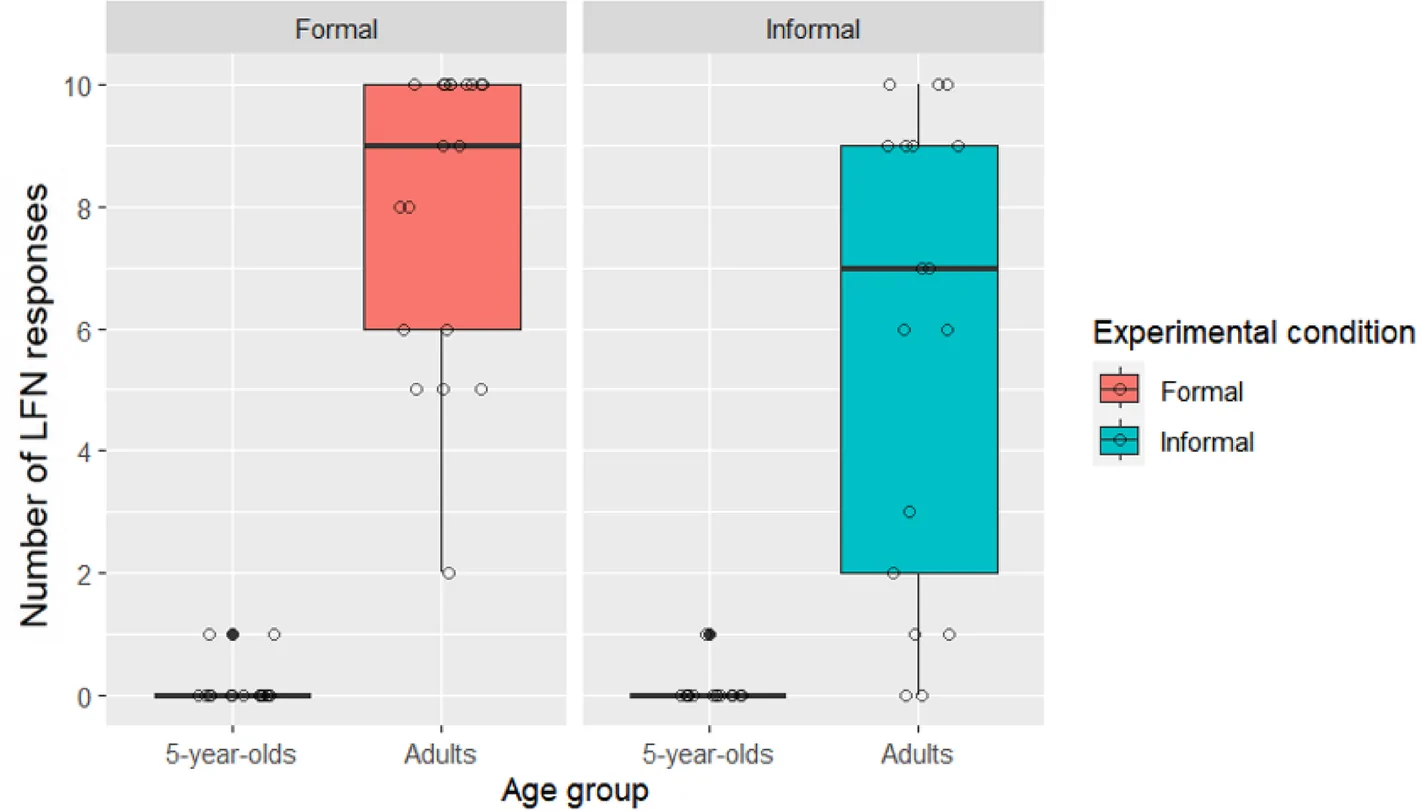
Boxplots of the number of Long-Form Negation responses produced by 5-year-olds and adults across experimental conditions. Each participant completed 10 target trials

**Fig. 2:**
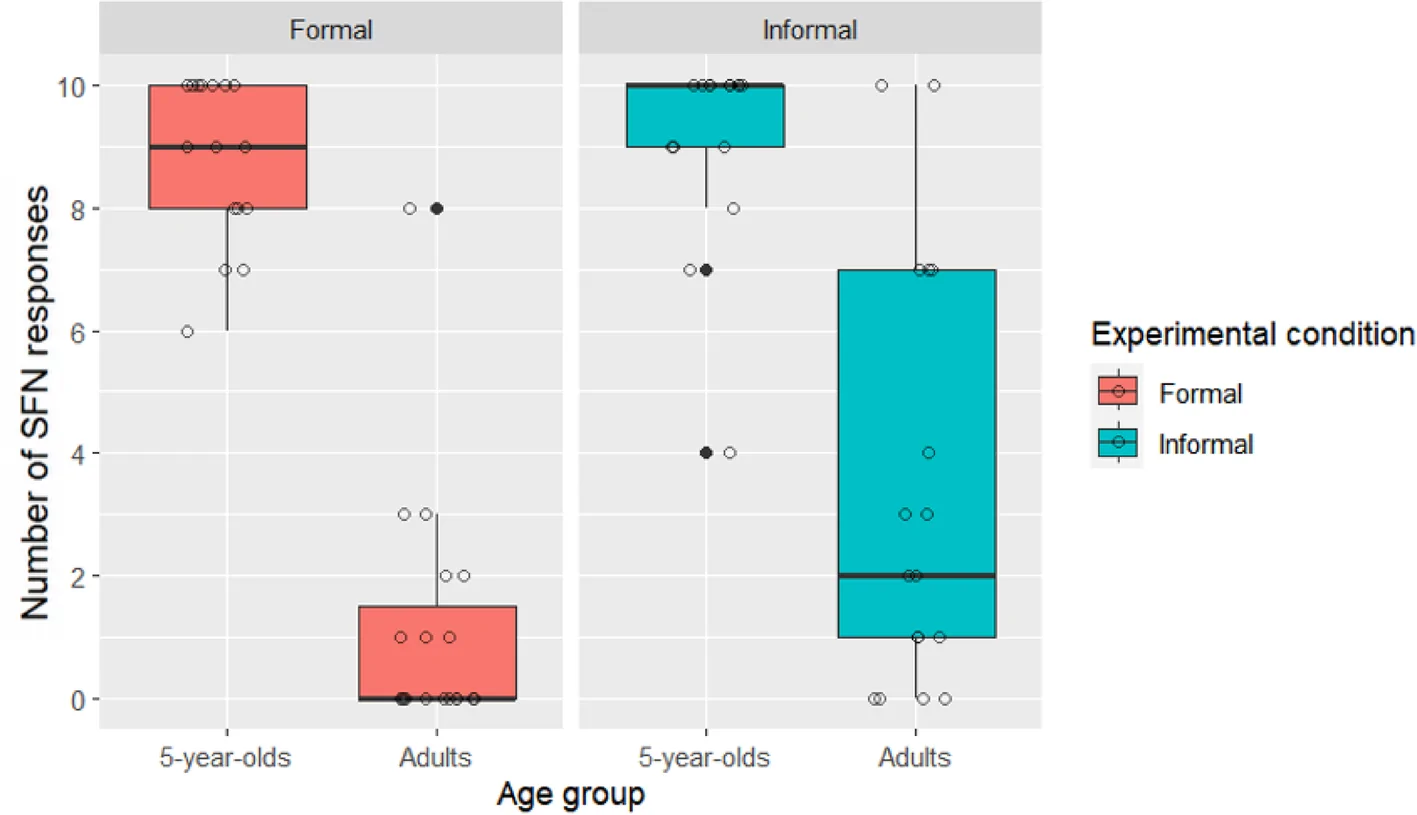
Boxplots of the number of Short-Form Negation responses produced by 5-year-olds and adults across experimental conditions. Each participant completed 10 target trials

Figures 1 and 2 show the distributions of long-form and short-form negation produced by adults and children across the two speech-style conditions. Descriptively, adults produced LFN more frequently in the formal condition than in the informal condition, whereas children predominantly produced SFN in both conditions.

Adults produced LFN an average of 8.05 times out of 10 target trials in the formal condition (*M* = 8.05, *SD* = 2.46) and 5.82 times in the informal condition (*M* = 5.82, *SD* = 3.81). Conversely, they produced SFN 1.11 times in the formal condition (*M* = 1.11, *SD* = 1.97) and 3.41 times in the informal condition (*M* = 3.41, *SD* = 3.48).

In contrast, 5-year-olds rarely produced LFN in either the formal (*M* = 0.12, *SD* = 0.34) or informal condition (*M* = 0.07, *SD* = 0.26). Across all participants, children produced only three long-form negation responses: two in the formal condition and one in the informal condition. Instead, they predominantly produced SFN in both the formal (*M* = 8.81, *SD* = 1.33) and informal conditions (*M* = 9.07, *SD* = 1.69).

Taken together, these descriptive findings suggest that adults adjusted their negation-form selection according to speech style, whereas 5-year-olds maintained a strong preference for SFN regardless of the speech-style condition.

To determine whether these descriptive patterns were statistically reliable, a generalized linear mixed-effects model was fitted (see Table 3). The results revealed significant main effects of speech style (β = 2.34, *SE* = 0.99, *z* = 2.37, *p* =.018) and age group (β = 9.35, *SE* = 1.52, *z* = 6.15, *p* <.001). The interaction between speech style and age group was not statistically significant (β = − 1.75, *SE* = 1.92, *z* = − 0.91, *p* =.363). In addition, a likelihood-ratio test indicated that adding the interaction term did not significantly improve model fit (χ^2^(1) = 0.79, *p* =.375).

Although the interaction was not statistically significant in the generalized linear mixed-effects model, planned pairwise comparisons using estimated marginal means were conducted because the interaction was of theoretical interest. The results revealed that adults produced significantly more LFN in the formal condition than in the informal condition (estimate = − 2.34, *SE* = 0.99, *z* = − 2.37, *p* =.018). In contrast, children showed no significant difference between the two speech-style conditions (estimate = − 0.59, *SE* = 1.65, *z* = − 0.36, *p* =.720), indicating that the frequency of SFN production did not differ between the two speech-style conditions.

Taken together, these findings indicate that adults adjusted their negation choice according to speech style, whereas children maintained a stable preference for SFN across both conditions.

**Table 3:** Generalized linear mixed-effects model predicting negation form selection

Predictor	β	SE	z	*p*
(Intercept)	−3.40	0.78	−4.36	< 0.001
Speech style (Informal vs. Formal)	2.34	0.99	2.37	0.018
Age group (5-year-olds vs. Adults)	9.35	1.52	6.15	< 0.001
Speech style × Age group	−1.75	1.92	−0.91	0.363

Random intercepts were included for participants and test items. Reference levels were the formal condition and adults. Likelihood-ratio test comparing models with and without the interaction term: χ²(1) = 0.79, *p* =.375

### Speech-Level Production

**Fig. 3:**
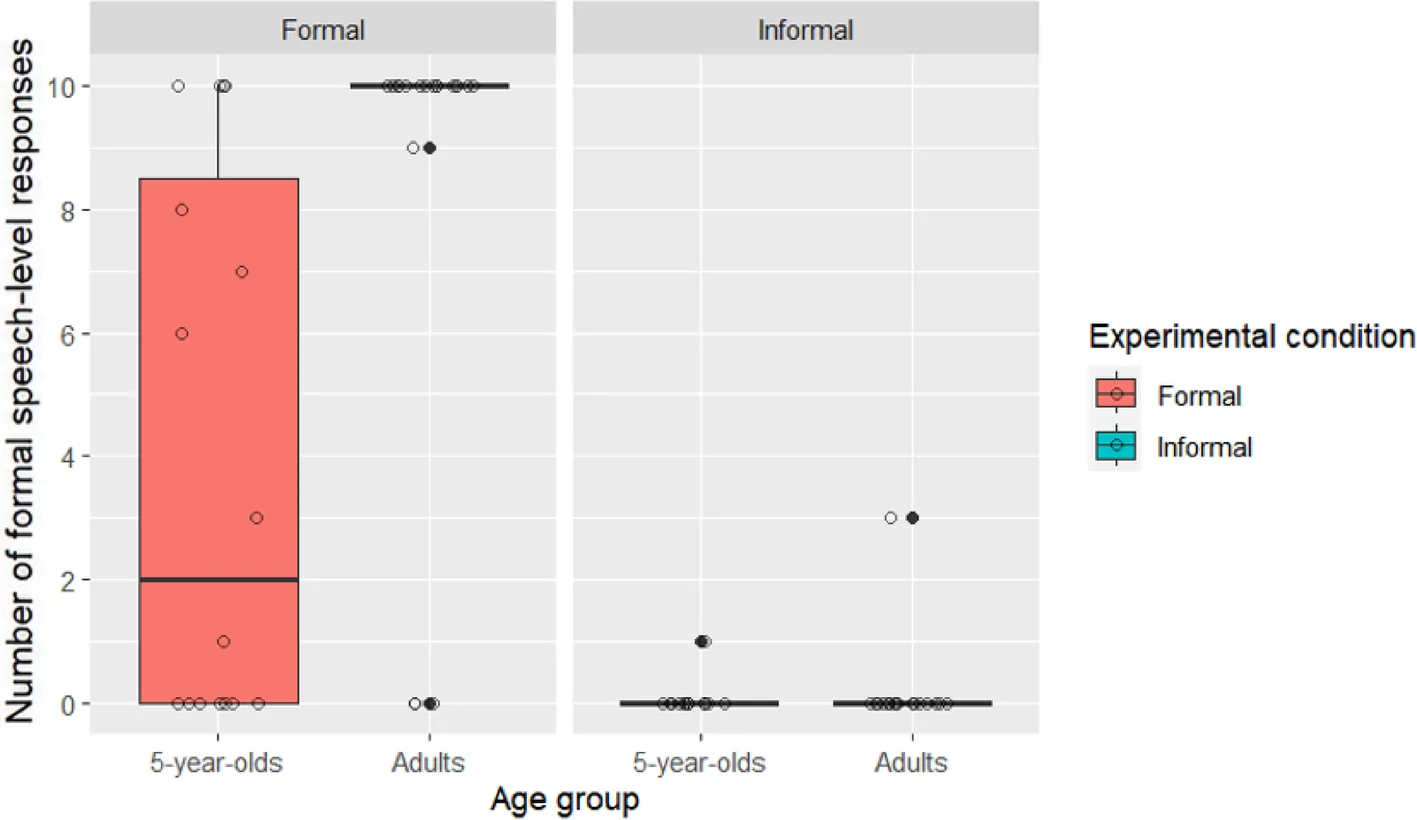
Boxplots of the number of formal speech-level responses produced by 5-year-olds and adults across experimental conditions. Each participant completed 10 target trials

**Fig. 4:**
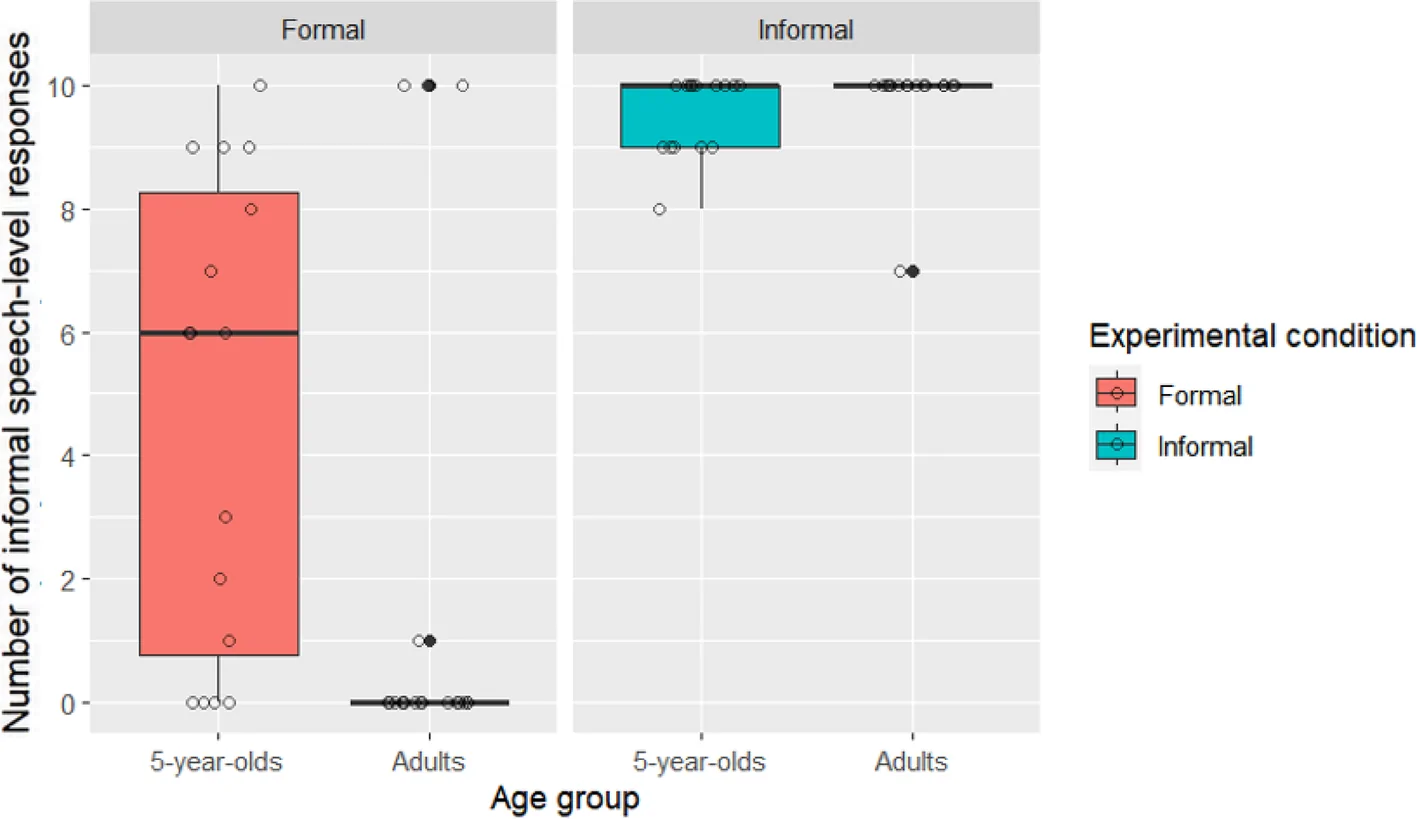
Boxplots of the number of informal speech-level responses produced by 5-year-olds and adults across experimental conditions. Each participant completed 10 target trials

For speech style, adults were shown to produce the speech style which matches the one which the puppet used in experimental conditions. Figures 3 and 4 depicted the distribution of adults’ speech level production by experimental conditions. Adults were likely to produce formal speech style in the formal condition (*M* = 8.37, *SD* = 3.73) and informal speech style in the informal condition (*M* = 0.19, *SD* = 0.75). Thus, they were likely not to produce informal speech style in the formal condition (*M* = 1.63, *SD* = 3.73) and formal speech style in the informal condition (*M* = 0.19, *SD* = 0.75).

Five-year-olds, interestingly, in the formal condition, chose to use a speech level that did not match the conversational partner’s speech level more frequently compared to adults. Figures 3 and 4 show that their production of formal and informal speech levels was more widely distributed in the formal condition compared to the informal condition.

Taken together, these descriptive findings indicate that adults consistently matched their speech-level production to the conversational context, whereas 5-year-olds showed greater variability, particularly in the formal condition.

### Children’s Production Errors

Though LFN was rarely produced in children, there is a possibility that children were not comfortable for producing SFN in formal condition if they relate SFN to informality and LFN to formality. To verify this possibility, we compared the frequency of children’s errors between the formal and informal conditions. We sorted out the errors children made across all the responses and classified them into five categories: unfinished sentences, yes or no responses without full sentences, sentences paraphrased in positive verbs, no response, and gramatically incorrect sentences.

Table 4 compared the number of errors across all the responses between conditions. We found that 5-year-olds made more errors in producing negation in the formal condition than the informal condition. The result of Welch’s t-test showed that the difference of error frequency in two conditions was significant (*t* = −3.69, *p* <.01). To be specific, they had more difficulty in finishing sentences and producing grammatically correct sentences and instead described animals’ behaviours with positive verbs to avoid using negation. This indicates that 5-year-olds hesitated producing SFN more in formal context than informal context.

**Table 4:** Errors children made in producing negation by experimental conditions (formal vs. informal)

	Formal condition	Informal condition	Example
1. Unfinished sentences	6	0	(English) “(The cow) does not sit down.” (Korean) “an anj-eu.”
2. Yes/No answers without full sentences	5	6	(English) “No.” (Korean) “a-ni-yo.”
3. Positive statements	9	5	(English) “(The monkey) is looking into the mirror.” (Korean) “geo-ul-eul bwa-yo.”
4. Nothing answered	2	1	
5. Grammatically incorrect sentences	4	1	(English) “(The snake) does not.” (Korean) “an hab-seub-ni-da.”
Total	26	14	

## Discussion

The present study examined whether speech style influences the choice of negation forms in Korean-speaking adults and 5-year-old children. Using a generalized linear mixed-effects model, we found significant main effects of speech style and age group. Adults produced significantly more long-form negation in the formal condition than in the informal condition, whereas children consistently preferred short-form negation regardless of speech style. Although the interaction between speech style and age group did not reach statistical significance in the mixed-effects analysis, the planned comparisons suggest that adults, but not children, systematically adjusted their negation choice according to speech style. Based on results, we claim that Korean negation implicitly expresses politeness and 5-year-olds are yet in the process of acquiring implicit meanings of negation forms.

Adults demonstrated a significant within-group effect of speech style. We found that formality of experimental condition predicted which negation form to produce. For adults, those placed in the formal condition were more likely to produce LFN than SFN, whereas those placed in informal condition were more likely to produce SFN than LFN. The finding implies that Long-Form Negation is preferred in formal situations to soften the level of assertion, and thus to be considered polite, whilst Short-Form Negation is more frequent in an informal communicative environment where a casual and firm attitude is allowed. This indicates the semantics of SFN, and LFN can be differentiated in terms of attitudinal meaning. This finding supports the arguments of previous research that the two forms of negation are not synonymous as LFN is more suitable for formal situations because it indexes polite attitude (Han, 2014) and formal style (Sohn, 2013). Although previous studies had raised the possibility that different pragmatic functions are embedded in the two forms of negation (Han, 2014; Sells, 2015; Sohn, 2013), this has not been tested in experimental conditions to show a causal relationship. Our study used an experimental design to verify the possibility that formality can constitute contextual grounds for addressers to select a negation form.

Our findings can be explained by the distinctive feature of Korean communication to prioritize politeness regarding interpersonal relationships. In Korean society, the relationships between individuals are highly cohesive and social hierarchy is valued, leading to restrain individual feelings (Gudykunst & Nishida, 1986; Kim et al., 1998; Sak & Shaw John, 2005). Due to these cultural features, researchers have classified Korean culture as HC culture (Hall, 1976) and Korea as a Confucian society (Brown, 2011) in which the social context plays a great influence on communication. In communication, Koreans try not to confront conflict directly and they express disagreement in a roundabout way to maintain social harmony, suppressing individuals’ self-expression (Kim et al., 1998). Due to these characteristics of Koreans’ communication, it seems that various forms of negation have been developed and used in Korea to show appropriate attitudes by context. Thus, Koreans consider a communicative context including interpersonal relations and an atmosphere of communication not only in taking a conversational attitude but also in choosing a grammatical form of expression which can deliver suitable pragmatic meaning. Therefore, the controversy on the difference between the two forms of Korean negation needs to be extended into pragmatic aspects in further arguments.

In contrast, children did not show a significant difference between formal and informal conditions. Five-year-olds could show the similar tendency of using SFN and, in contrast with adults, seemed not to distinguish negation forms based on the speech style of the conversation. We surmise that the speech styles expressed by verbal endings were acquired first at an early age and they will be fully linked to syntactic forms of negation later in development. In addition, we propose the possibility that the informal speech style and SFN are linked to each other earlier than formal speech style and LFN. To our knowledge, this is the first empirical evidence to show the initial stage of the development of Korean children’s ability to relate speech style to negation form. More detailed information on how Korean children completely connect speech style and negation from after age five should be examined in further studies.

The planned pairwise comparisons demonstrated a significant effect of speech style in adults but not in children. Thus, adults systematically adjusted their choice of negation according to speech style, whereas preschool children showed little evidence of such pragmatic adaptation. This pattern is consistent with the view that pragmatic sensitivity to speech style continues to develop beyond the preschool years.

It was shown in our study that children highly preferred using Short-Form Negation and that Long-Form Negation was rare in children’s utterances. This finding is similar to those of previous studies that suggested Long-Form Negation was infrequent in young children’s speech (Lee, 2009; Kim, 2003, 2014), and overgeneralization of SFN was found in a two-year-old child (Kim, 2014). Though our findings on this phenomenon are scarce, it seems possible to argue that the result of this study gave supporting evidence that overgeneralization can occur in older ages. However, based on the results of previous studies that Korean children acquire LFN before age four (Chang et al., 2014; Cho & Hong, 1988; Choi & Zubin, 1985; Lee & Im, 2003), the mismatch between its acquisition and rare usage, the phenomenon which Kim (2014) called “overgeneralization”, is still highly striking.

We offer two possible explanations for this mismatch. Firstly, as it appears more complex contextual meaning is embedded in LFN, the usage of LFN may not in itself reflect a deeper understanding of its complex contextual meaning. Second, even if young children do have semantic knowledge of LFN, children can value the economy of negation use over the pragmatic meaning of it.

To elaborate further on the first point, although 5-year-old children can produce LFN, they may replace it with SFN because of limited semantic knowledge on LFN and limited ability to add their active inference in processing it. The phenomenon of young children’s overgeneralization of SFN was documented in a previous study (Kim, 2014), yet the study was limited in that the origin of the phenomenon was not specifically described. Kim argued that young children highly preferred using SFN to LFN because it is easier to produce and can communicate meaning more clearly. Considering the arguments from previous literature, it can be inferred that children’s overgeneralization of SFN may stem from LFN having more layers of meaning than SFN. Thus, LFN could require a greater cognitive burden for young children in processing. Studies in support of relevance theory or contextualist accounts have argued that the suffix “-ci” of LFN is metalinguistic use of language, requiring active pragmatic inference to process (Noh, 2000, 2016), and contextual information needs to be supplemented for SFN to acquire a similar level of sensicality in metalinguistic negation (Carston & Noh, 1996; Noh et al., 2013). Based on these arguments, LFN may have, in addition to the literal meaning, more complex semantic content than SFN which needs to be processed based on active cognitive inference. Hence, it can take time for young children to fully understand and frequently use LFN even after they have initially started to use it. Considering the result of adults in the current study, formality could be one part of the semantic content to be considered in processing LFN. In summary, young children can rarely produce LFN before they fully understand its deeper meaning and make inferences on it based on context, even if they have already acquired the literal or propositional meaning of LFN.

Regarding the second explanation that even if young children do have semantic knowledge of LFN, it is possible that they chose to produce a more efficient form of negation regardless of semantic meanings in order to communicate the literal meaning of negation. In other words, 5-year-olds can value the economy of using SFN over the complexity of using LFN because of their lack of knowledge on pragmatic effects of various forms of negation. Children showed the least difference from adults in SFN production in informal context, which suggests that using SFN with informal speech ending is the most fundamental and efficient way to communicate the literal meaning of negation. Therefore, it seems that the ability to use diverse negation forms based on pragmatic contexts develop later than the acquisition of linguistic forms. This explains why children’s use of negation sounds childish and can be distinguished from adults’ use.

These two claims can also be supported by our finding that children made more errors when trying to produce negation in formal condition than informal condition. This means that producing negation in formal contexts is accompanied by a cognitive burden for children. Notably, among various types of errors, failure to finish sentences occurred most frequently in the formal condition but did not occur in the informal condition. The results show children who have acquired SFN but only have a shallow understanding of complex semantic meanings of LFN may feel uncertain about producing SFN in formal contexts to communicate politeness, increasing the cognitive burden to process negation. Even if they have some understanding of LFN, they could avoid producing LFN as it involves the cognitive burden of making pragmatic inferences and verbalizing more grammatically complex sentences.

On the other hand, the current study revealed verbal endings can constitute a pragmatic context in which listeners are demanded to take an appropriate attitude and select a negation form. Although previous studies argued that formality of speech style could influence the choice of negation forms (Han, 2014; Sohn, 2013), they focused not on specific verbal endings but on formality or informality of situations on an abstract level. The experiment on adults showed that negation selection can be changed when only verbal endings were manipulated. This result is meaningful as it verified the relation of formality and negation at a practical and specific level.

In light of the evidence, this study shed light on the possibility that the pragmatic development of children in Korean culture is distinctive. The social pressure to show politeness based on a communicative context can lead Koreans to use a more inefficient form of negation, LFN, for the sake of achieving complicated pragmatic goals. Korean children have to learn to consider communicative contexts, such as formality of speech styles, the relative social ranking of conversational partners, and the atmosphere of conversation to choose proper grammatical forms in communication, which may not be an important issue for children of individualized cultures (Gudykunst & Nishida, 1986). Thus, young Korean children in the process of learning the polite use of language can have difficulty of using LFN, even if they already acquired the linguistic form itself. Further cross-linguistic study is needed to reveal the differences in the effect of formality on negation use of children from Korean culture and other cultures.

One limitation of the present study is the relatively modest sample size, particularly for detecting interaction effects in mixed-effects models. Because generalized linear mixed-effects models appropriately account for repeated observations within participants, they provide more conservative estimates than conventional logistic regression. Future studies with larger samples will be able to examine developmental differences in speech-style sensitivity with greater statistical power.

Although the current study provided evidence of the effect of formality on the selection of negation forms, the results cannot be generalized to age groups other than adults in their 20 s and 30 s and 5-year-old children. As it is probable that the pragmatic aspect of negation can be acquired in the middle of childhood, future research comparing the results of experiments on various age groups beyond age five can provide more profound information from a developmental perspective. It will be necessary to verify through future research the various possible causes for the overgeneralization of SFN in young children that were proposed in this study. In addition, various factors relating to language development like parent’s SES (socioeconomic status), type of family, the experience or education of a second language, and other cognitive abilities need to be considered in future research.

Additionally, the findings of this study can be limited in applications of real-world conversation. This study tried to make the conversational situation between a puppet and participant as natural as possible by using a Bluetooth speaker inside the puppet. However, there is a possibility that some participants realized that the puppet was not an interactive conversational partner. This could have led participants to produce utterances repetitively without processing contextual information as thoroughly as they do in spontaneous language production in the real world. Also, in the experiment, the personal information about the puppet, such as age and social ranking, being ambiguous could have caused the differences between the participants’ utterances in experiments and those in the real world. Thus, further studies are needed to investigate if the results of this study can be replicated in conversational settings with a real and interactive person and if children and adults’ negation selection changes depending on the features of conversational partners in the contexts of real-world communication.

In conclusion, the present findings suggest that adults adapt their choice of negation forms according to speech style, whereas 5-year-old children continue to rely predominantly on Short-Form Negation regardless of speech style. Although the interaction between age group and speech style was not statistically significant in the mixed-effects analysis, the within-group comparisons indicate that pragmatic sensitivity to speech style is evident in adults but has not yet fully emerged in preschool children. The study demonstrated that Korean adults use negation forms depending on formality of a communicative situation, the factor that had not been directly studied in other research on negation. Various forms of negation may be used to enrich communication in consideration of context, despite the fact that they share the same propositional meaning, supporting the account that Korean syntax can be pragmatically and contextually decided (Kiaer, 2014). In addition, the results of the experiments revealed the depth of Korean-speaking children’s knowledge of negation forms and provided possible explanations for the reason why Long-Form Negation is more difficult to produce. It also showed that interpersonal attitude is one of the key factors in children’s language development in Korea. This study offers a comprehensive approach encompassing pragmatics, semantics, and syntax, contributing to research on negation.

## Appendix

### Experimental Items (Translated into Korean with Formal or Informal Verb-Endings)

1. Does the bear eat an apple?
2. Does the rabbit sit on a chair?
3. Does the cow write?
4. Does the milk cow drink water?
5. Does the monkey look into a mirror?
6. Does the horse brush hair?
7. Does the hen swim?
8. Does the tiger put on clothes?
9. Does the pig pick up the phone?
10. Does the snake say hello?

### Filler Items

1. How many dogs are there?
2. Does the cat watch TV?
3. Does the turtle clean up?
4. Does the elephant drive?
5. What does the squirrel hold?
6. Does the deer get a shot?
7. How many fishes are there?
8. Does the rhino kick a ball?
9. What color is the sheep’s hair?
10. Where is the boy sitting?

## Footnotes

1 By speaking in non-honorific styles, a speaker switches from the half-talk style to the plain style to communicate the new information which is particularly noteworthy to the listener or to the negative predisposition to the information (Lee, 1991).
